# Book Reviews

**Published:** 1806-09

**Authors:** 


### CRITICAL ANALYSIS

OF THE

### RECENT PUBLICATIONS

ON THE

### DIFFERENT BRANCHES OF PHYSIC, SURGERY, AND MEDICAL PHILOSOPHY.

### *Dr. Pinckard's Notes on the West Indies.*

[ Continued from p. 561—563. ]

The yellow fever, as it is generally called, appears to have engaged our author's particular attention. He seems to have omitted no opportunity of ascertaining its true character; and although he does not pretend to have discovered either its prevention or cure, we think he has suggested several useful hints respecting both these important objects; and his account of the symptoms, progress, and causes of this destructive scourge appears to us to be particularly accurate. Soon after the capture of Essequibo and Demarara this disorder made its appearance among the troops. The first soldier that was attacked, died on the sixth day after his admission into the hospital.

"The body was examined," says Dr. P. "with a view to ascertain the changes produced by the disease, but the appearances were not precisely such, as, from conversing with other practitioners, and reading a variety of authors, we had been led to expect. The stomach was found to be the organ which exhibited the strongest marks of derangement. The inner coat was surcharged with blood, appearing very red, and at one spot near the upper orifice it was of a livid hue, and its texture so weakened, that the finger was passed through it by only a slight pressure. I sincerely hope that frequent opportunities may not occur, but if, unhappily, they should, we shall avail ourselves of them, in the hope of ascertaining, correctly, what are the diseased appearances caused in the different viscera by this fatal malady.

"The name commonly given to this disorder would seem to be highly inaccurate. Our patient, although several days ill, had no yellowness of the skin, until a few hours before he died. If, therefore, his dissolution had occurred only a short time sooner; or if he had recovered previous to the period when this change of colour took place, we could not with any correctness have called the fever he had suffered a *yellow* fever, although he had undergone all the characteristic symptoms of the disease, so termed, except the casual one from which it has been improperly named. Moreover, affixing to a disease a name derived from a symptom, and particularly a symptom which is not always present, is calculated to deceive, and may be of dangerous tendency, by rendering the pracpractitioner unsuspicious of the real nature of the disorder, until it is too late to check its destructive progress."

In a short time afterwards, Dr. P. had another opportunity of inspecting the body of a soldier who had fallen a sacrifice to the same disease.

" The appearances," he says, " were similar to those I had witnessed in the former case, except that, in the present instance, there were some striking marks of inflammation within the thorax. I shall further avail myself of the painful occasions that may occur, during my stay in this colony, in order that we may compare the cases with such as may be examined at Demarara during my absence, and after my return, in the hope that we may, thereby, be enabled to judge, with more accuracy, regarding the general appearances, and the actual changes induced by the disease.

" Since I wrote to you last we have had the misfortune to lose one of the soldiers by the disease called coup de soleil, or ictus solis. The poor man was on guard, and stationed as centinel to take his turn of duty for two hours at the gate of the fort, but before this short period had elapsed he was suddenly seized, fell down, and expired at his post. The day was excessively hot, and the perpendicular rays of the sun fell in their fullest ardor upon his head. Others of the men have likewise experienced the disease, but we have only, in this instance, seen it fatal.

" I may also mention to you a very remarkable and melancholy case of yellow fever which has occurred at fort William Frederic, affording a striking example of the nature of this afflictive malady. A grenadier, named Llewellyn, a handsome and well made man, of a robust figure, was brought into the hospital, complaining only of an uneasy sensation about the region of the stomach, which although indescribable, conveyed to him an early assurance of the fatal tendency of the disease, with which he was attacked. On asking him to explain his complaints, and the feelings which so alarmed him, he replied—" *I feel that I shall die*," and placing his hand over his stomach, said he had " something there" that would soon kill him. We used all the means in our power to divert him from this desponding impression—but in vain! nothing he said could restore him, for he felt the hand of death upon him. He evidently laboured under an attack of the disorder, termed yellow fever; but, with the exception of this fatal sensation, no symptom was present which could have led to the apprehension of immediate danger. At my next visit on the following morning, I found him sitting up, and apparently somewhat relieved; but on my asking him how he was, he still replied—" *Dying! I feel that I shall soon be gone.*"—He continued perfectly rational and collected until the next day; when, alas! death but too surely confirmed the accuracy of his predictions. From the moment of attack he had felt assured, that nothing could save him! The indescribable feeling he complained of, seemed to induce a rapid exhaustion of the vital powers, while it formed, not only the leading feature—but almost the only symptom of the disease.

"Upon examination, we found the same redness of the inner coat of the stomach, which we had observed in all cases of the yellow fever, and in the lungs were some strong marks of recent inflammation."

About a month afterwards, when the Author's observations had been more diversified, he says:

"Not to fatigue you with a minute detail of the appearances, I may briefly observe, that in the stomach they have hitherto been uniform, but in the other viscera very uncertain and dissimilar. With respect to the symptoms of the disease, we begin to discover much instability. Either vomiting, low delirium, singultus, or coma, with or without yellowness of the skin, forms the prominent feature—each in its turn seeming to give the character of a distinct malady—but all terminating, within a few days, under the usual appearances assumed by our great common destroyer."

The following case shews the rapid fatality of this terrible disease: it occurred in the month of December.

"A few days ago, on my passing by the guard-house, I observed a grenadier, named Chapman, sitting among the men who were upon guard, seemingly unwell; and on my asking him if he was sick, he answered in a firm strong voice, that he felt "*a little poorly with head-ach, but not ill;*" still I perceived that he had more of indisposition about him, than he was aware of, and I was the more particularly led to notice any symptoms of disease in this man from his having been repeatedly an object of conversation and remark, in health. He was a hardy, robust grenadier, and from having been much exposed on fatigue-duty in Ireland, during the time of the sickness which the troops had experienced at Spike Island and at Cove; from having supported much of similar duty on the passage; and also a considerable share since his arrival in the West Indies, without suffering from it, the officers had pointed him out to me, as a person who was "secure against *even yellow-fever, and the doctors.*" He was sitting in his usual clothing, talking to the men of the guard, sensible only of slight head-ach, and lassitude: but as I saw evidently that it was an attack of fever, without mentioning this to him, I hinted that it would be better he should go to the hospital, if he was at all unwell, in order that he might have the benefit of medical attendance, and of such remedies as his case required. He instantly expressed great alarm, and said, "*I am not ill: if you take me to the hospital, I shall catch the fever and die.*"—On my stating the impropriety of his remaining among the well men, and not using the proper means of recovery, he replied, "*I am not sick, and only want an appetite to be quite well;*" and when I urged him further to go into the hospital, he answered with quickness, "*Indeed I am not bad, and if I was, I would rather stab myself at once, than go where so many are dying every day of yellow fever.*" Poor fellow! he was wholly unconscious, that the disease, he so much dreaded, was upon him—and as I found that his terror of the hospital was quite insurmountable, I did not augment his alarm, either by insisting upon upon his being carried thither, or by telling him that the fever had already seized him; but in order that he might be removed from the guard-room, I gave directions for a hammock to be put up under the piazza of the house, where he might be in quiet and entirely alone. This being done, without delay, I prevailed upon him to go directly and lie down, and was glad to see him safe in bed; for, in my conversation with him, I had discovered enough to cause strong apprehensions lest he should die amidst the men of the guard, before we could have him removed! Although he felt so little of illness, and those around him were so wholly insensible of his peril, there was an indescribable something about him, particularly in his countenance, which bespake extreme danger; and from which the eye of experience might see that he was soon to die! After having him placed in the hammock, and prescribing for him such remedies as were necessary, I instructed the medical gentlemen, who had lately arrived at this post to give me assistance, to pay particular attention to his case, remarking, that from his present appearance, I should not be surprised if, in the course of an hour or two, I should learn that he was dead. The event verified my suspicion: a convulsive tremor of the muscles quickly seized him, and at noon he was a corpse! Such are the fevers of this climate! Often a man is well in the morning, and at night is no more: nor is it possible for any one, who has not seen many cases of the disease, to judge of the degree of danger which threatens those who are attacked."

---

*Surgical Observations, Part the Second, containing an Account of the Disorders of the Health in general, and of the digestive Organs in particular, which accompany local Diseases, and obstruct their Cure: Observations on Diseases of the Urethra, particularly of that Part which is surrounded by the Prostate Gland; and Observations relative to the Treatment of one Species of Nævi Materni.* By JOHN ABERNETHY, F.R.S. &c. 8vo. 1806.

It always gives us pain if we object to the honest endeavours of enlightened men. No one can doubt the good intention or abilities of Mr. Abernethy; nor can it be questioned for a moment, that much useful information may be drawn from whatever he offers to the public. But it is impossible not to admit, that there is sometimes a degree of crudity in his performances, which is neither consistent with his large advantages, nor the length of time he has appeared as an author.

In the work before us he seems, by the motto he has chosen, to apologize for undertaking a department rather medical than chirurgical. For our own parts, we feel so thankful for information from whatever source, that we never scruple to attend to it; but it must occur to every one, that what may appear new and extraordinary to a surgeon, may be familiar to a physician, and vice versâ.* Thus Mr. Abernethy was probably more surprised than

* See a Communication by Dr. Brocklesby, Med. Obs. & Enq. Vol. iv. p. 371.

than several of our Readers would have been, at discovering that many nervous complaints arise from hepatic affections; and that derangement of the organs of digestion would produce all the symptoms of pulmonic disease.

The first Chapter is "on those disorders of the system in general, and of the digestive organs in particular, which accompany local diseases, and which, whether they be sympathetic or idiopathic, considerably obstruct the cure of these diseases." We shall transcribe the words of the Author, to convince the Reader of our impartiality.

"An evil seems to me to have arisen from the artificial division of the healing art into the medical and surgical departments.—This division has caused the attention of the physician and surgeon to be too exclusively directed to those diseases, which custom has arbitrarily allotted to their care. The effects of local disorders upon the constitution, have, in consequence, been too little attended to; and indeed I know of no book, to which I can refer a surgical student for a satisfactory account of those febrile and nervous affections, which local disease produces, except that of Mr. Hunter. The reciprocal operation of constitutional disorders upon local diseases has obtained still less attention. To investigate more particularly some parts of these subjects, and to bring them forward to public notice, are the proposed objects of the present paper.

"No part of the animal body can be very considerably disordered, without occasioning a correspondent derangement of the whole constitution. Such disorder has been considered by Mr. Hunter as the result of universal sympathy. This consent of the whole constitution with its parts, manifests itself, in particular instances, by a greater disturbance of the functions of some organs than of others; and from this circumstance, these diseases have derived the appellations by which they are commonly distinguished. If the actions of the sanguiferous system be principally disturbed, and the temperature of the body subject to unnatural variations, the disease is termed fever: if the nervous system be chiefly affected, a state of vigilance or of delirium may be produced: convulsions and tetanus take place when the muscular system is more particularly disordered. Though the especial disorder of particular organs thus gives a character and denomination to the disease, it is sufficiently evident, in every instance, that the whole constitution is disturbed, and that parts of it are chiefly affected, perhaps from unknown circumstances relative to the nervous system, or it may be from a predisposition to disorder existing in the parts which are chiefly affected. It seems to be ascertained, that persons of particular constitutions are predisposed to those febrile actions of the sanguiferous system, which constitute the inflammatory fever; that there is a propensity to convulsions in children, and to tetanus in the inhabitants of warm climates.

"It

" It may be a fit subject for enquiry, whether it be possible for particular organs to become affected otherwise, than through the medium of the nervous system in general. Though some instances of sympathy are strange, and perhaps inexplicable, it must, I think, be admitted, that the inflammatory fever, the state of vigilance and delirium, convulsions and tetanus, which arise in consequence of injuries of the limbs, are produced by irritation imparted to the brain, which, by a kind of reflected operation, occasions a greater disorder of some organs than of others, and thus gives a character and denomination to the disease.

" That the stomach and bowels are disordered by injuries and diseases of parts of the body, has been remarked by various persons; but the subject has never been extensively surveyed, nor viewed with that accuracy of observation, which its high importance merits. It has been observed, that sprains of tendinous or ligamentous parts produce sudden sickness; and Mr. Hunter has attributed that shivering which is consequent to accidents, and attendant on some diseases, to the state of the stomach. It is also known that, in some local injuries from accident or operations, the stomach has appeared to be the part principally affected. But these remarks have been made only in a cursory manner; and it is my intention to examine the subject more particularly. The connexion of local diseases with the state of the constitution in general, also appears to me either not sufficiently understood or not duly regarded by the generality of practitioners; and to this subject I also mean to claim their particular attention. I shall in the first place select two cases, to shew how the stomach and bowels, or to speak yet more extensively, the digestive organs, may be affected from local disorder."

A Case follows, of a gentleman on whom the operation was performed, for an adherent omental hernia. The consequences of the operation, though conducted with the utmost caution, were great disorder of the constitution, with a full and strong pulse, furred tongue, great anxiety, restlessness, and total want of sleep. The stomach was particularly affected, being distended, uneasy on compression, and rejecting every thing that was swallowed. In this situation the patient very wisely refused to take any thing, excepting his remedies and water, till the irritability of his stomach ceased; after which all the symptoms subsided, and his medicines produced their proper purgative effect. We have chosen to place the events in this order, because we cannot ascribe the patient's relief to the operation of the remedies, but the operation of the remedies to the gradual cessation of the high irritability produced by the new state of certain parts. In the same manner we conceive Mr. Abernethy might easily explain the progress of other complaints after operations which he describes in a Note. " In these cases, (says he) opium fails to quiet the irritability of the stomach, and I have always considered it a primary object to produce secretions into the bowels, as I have observed, that if discharges can be produced per anum, the stomach becomes tranquil." That is, we should say, as the disease ceases the bowels obey the stimulus of the remedies exhibited.

Another Case follows, in which the stomach was violently affected by a local injury; and the author very truly observes, that such cases are very common. The stomach and brain are organs more easily affected than any other, and sympathize with almost every part of the body. This sympathy, in children, may often arise from teething; but our indolence is so apt to impute almost all diseases in infancy to this cause, that we think it was unnecessary to add to this too general resource among practitioners of most descriptions.

After this the author, without adverting to any particular disease, enters into a discussion of those nervous complaints which arise from a want of digestion, or a slow action of the organs subservient to that office. Among the diagnostics he points out, we are forced to admit that many are very fallacious. The colour of the fæces varies much in people who are in health, and how pale soever they may be one week, if no symptoms of ill health appear, we should not teach a patient of this description to concern himself about their complexion. The urine is still more uncertain, varying from day to day, nay from time to time, whilst no perceptible change appears in the state of the subject. We cannot omit a few other remarks in this passage. "The cuticle of the tongue (says our author) sometimes seems to have lost its transparency, and to become permanently white in consequence of continued irritation." In this passage is there a proper distinction made between a furred tongue and a tongue naturally white? Does not that member vary in its complexion like the gums, which assume every shade from red to white coral?

"The extent of the power (it is added) which the intestines possess, of converting substances contained in them into chyle, or of preventing chemical changes, is unknown. It is probable that much unassimilated matter is absorbed by the lacteals, when the digestive powers fail in their functions. This is *demonstrably* the case in diabetes, where the vegetable matter floats in the serum of the blood, rendering it turbid, and afterwards combines so as to form sugar in its passage through the kidnies." We confess all this has not been demonstrated to our satisfaction.

Some good practical reflections follow, on the probable progress of chylification, and the office of the bile; but they are either not new, or not satisfactory. The author expresses his surprize, that when, during the slow progress of cancer or lumbar abscess, the various digestive organs have appeared deficient in their functions, he has not found any real disease in those parts by examination after death. But we should recollect, that in most tedious illness the patient finds himself incapable of any kind of application. Why then should we be surprised that the stomach or liver, or any other part, should at such times imperfectly perform its its office any more than the brain? "It naturally excites surprize, (continues our author) that such a state of irritation and imperfect performance of the functions of those parts should exist for so long a time, as in many cases it is known to do, without producing organic disease; still I believe it may be set down as an axiom, and which will be verified by every observation I have made, that a state of irritation naturally leads to those diseased actions which produce an alteration of structure in the irritated parts." We sincerely wish this paragraph had been illustrated with some well marked cases, that had occurred often enough to induce so decided a mode of expression. The instances that follow, in which an alteration of structure was proved to exist, are cases where the intestines appear to have been primarily affected. Immediately after the above position too, we have an account of that class of diseases which are generally termed nervous, arising from anxiety, improprieties of diet, a sedentary life, or over exertions of any kind; at the close of which we are told, "it is indeed no wonder that the continual irritation of our unnatural diet should by degrees produce such disorders of the digestive organs." This is certainly very true; but we do not immediately see, how the remarks are connected with local diseases. That local will produce constitutional disease no one ever yet doubted, and also that a general good state of the constitution is often necessary for the cure of local complaints. It might therefore, we think, have been sufficient for the author to remind us of some strong cases, in which inconveniences had followed an inattention to such events, without the necessity of pursuing the subject for so great a length. If this had been thought necessary, we cannot help wishing, for our relief and the advantage of the reader, that more attention had been paid to arrangement, so that we might at least have known what we were to expect in a chapter by its superscription. It is not our intention to insinuate, that Mr. Abernethy's labours are useless; the work abounds with valuable suggestions, and the concluding cases are well selected, to show that a general ill state of health, arising from any cause, may counterfeit local disease.

The next Section contains Cases, with remarks, in which local disorders of the head, produced by blows, are kept up and aggravated by affections of the digestive organs. In this we meet with many good practical hints.

The third section is on diseases of the throat, skin, and bones, which so much resemble venereal complaints that they are frequently treated as such, but which take place without any reasonable ground to attribute them to the absorption of any morbific poison. The first was one of those sore throats which Mr. Hunter has described as sometimes mistaken for venereal, but which he remarks could not be such from the ulceration not being regularly progressive till mercury was exhibited. Another case is related, which in consequence of copper spots, was too hastily pronounced

 venereal venereal by others, but which Mr. Abernethy cured by attending to the chylopoietic organs. Two others follow of thickened periosteum in those bones which are most frequently affected by the venereal virus. These however, by the manner in which they gave way to remedies directed to relieve the stomach and other viscera, were proved not to be syphilitic. Some judicious remarks follow these cases.

The fourth section is more miscellaneous in the nature of the local diseases which were relieved by attention to the digestive organs and to the general health. Many of the cases were indeed such as might be expected to yield to such treatment, being those indolent ulcers which are sometimes called scrofulous. Others were ill-conditioned ulcers, depending sometimes on poverty, bad treatment, or a local irritation, by degrees become habitual or chronic. These were all relieved by changes produced in the constitution. Two more important cases follow, much resembling cancerous breasts, which were relieved in a similar manner. This section concludes with the following remarks.

"Before I had paid attention to those complaints which arise from, or are aggravated by constitutional causes, I could not have believed that such considerable local diseases, after resisting various topical and general means, should give way so readily and completely to small doses of medicine. It is only by considering the manner in which this effect is produced, that the subject can be placed in a proper point of view.

"An attention to the state of the bowels is indispensably necessary, even in the common practice of surgery. A simple cut of the finger frets into a bad phagedænic sore, which resists every local remedy so long, that amputation is at last proposed. This is the consequence of bad health, which in its turn is aggravated by the irritation of the sore. The patient has a furred tongue, with other symptoms of disordered digestive organs. An attention to this disorder corrects the painful state of the sore, which now heals rapidly under simple dressings.

"A patient has a disorder in the urethra, almost too trivial for surgical attention; yet producing much inconvenience. The functions of the digestive organs are impaired, and he is hypochondriacal. He consults a physician, under whose care, his general health is amended, and he no longer feels or thinks of the local disease.

"An erysipelatous inflammation of the leg is imputed to some trivial cause; as for instance a gnat bite. It becomes worse under the common remedies. The health has been long declining, and the chylopoietic viscera are obviously deranged. The erysipelas is quickly cured by medicines prescribed for that disorder.

"A patient supposes that his knee is strained; for pain and inflammation of the joint suddenly come on, with deposition of fluid into the articular cavity; this attack is attended with fever, furred tongue, and unnatural discharges from the bowels. Leeches, cooling cooling washes, and poultices, in short, all topical applications are unavailing. It is a case of rheumatic inflammation, for which a physician is consulted. Five or six weeks elapse without any abatement of the disease, the patient being almost unable to stir in bed. An alteration in the health suddenly takes place; the tongue becomes clean; and there is no longer any pain in the knee. All the fluid is absorbed from the joint in two days, and the patient walks about his chamber. Or there may actually have been some local injury; but the consequences are very considerable and violent, and quite incommensurate to the cause. Such occurrences can only be assigned by imputing the effects to the state of the health in general. I could relate a great number of cases to illustrate this subject, but it does not seem to me to need any further exemplification.

"I again repeat, at the conclusion of this section, that though I admit the possibility of the existence of diseases strictly local, and have adduced some instances of them, I consider the diseases, which I have been describing, to arise from disorder of the health in general, which is often caused, though sometimes merely aggravated, by disorders of the digestive organs; and it follows, if this view of the subject be correct, that such diseases may sometimes exist, without any manifest disorder of the digestive organs. The disorders of these viscera may act in a two-fold manner on the constitution; they may be the cause of an impure or imperfect state of the blood, and they may cause or aggravate nervous irritability. Whether in consequence of such effects they are to be regarded as the predisposing, as well as the exciting causes of such diseases as I have described, is an enquiry very worthy of investigation; but it does not appear to me to be determinable by the facts which have been recited."

The fifth section is on disorders of parts which have a continuity of surface with the alimentary canal.

"I had formerly observed spasmodic strictures of the œsophagus to disappear under various modes of treatment, in a manner which I did not understand. Mercury seemed to effect the cure in three instances. Many cases have occurred to me lately, in which the irritation in the œsophagus seemed to be first excited and afterwards maintained by disorder of the digestive organs. It will be readily allowed, that spasmodic strictures of the œsophagus, when long continued, may cause a thickening in the affected part of the tube, and thus the stricture may become permanent. One instance will be sufficient to illustrate and verify this view of the subject; indeed, I merely wish to excite attention to this subject, for I am incompetent to give an opinion as to the frequency or degree, in which affections of the stomach produce these disorders."

The cases which follow are very numerous, but we cannot feel satisfied that they all arose from a continuity with the alimentary canal, or were at all affected by remedies directed to those parts. That a case of ozæna should be suspended during a bilious attack is

 quite quite analogous to what we often see of other local diseases, which for a time seem to cease on the access of any constitutional complaint. We should feel equally sceptical of the gentleman's case whose *pseudo-syphilitic* symptoms and deafness were relieved by a two months course of pil. hydrargyri, however gentle. Such a course has relieved so many chronic diseases of various descriptions that we see no reason to suspect that the cause of the disease was to be traced to the digestive organs, or that the cure should be imputed to the relief *they* experienced from the remedy. Indeed we are not certain, in this instance, what Mr. Abernethy's opinion of the case was, when he remarks that the digestive organs were disordered by the *effect of the poison.**

The subsequent remarks on ophthalmia contain nothing new.

Some cases follow, of diseases in various parts of the skin. That the skin is continued from the external surface over the whole intestines, we shall not dispute; but a chronic ophthalmia, or an herpetic state of the leg, if connected with the stomach, we should rather say was kept up by a general want of health, than by a continuity of surface from the intestines to those parts.

The sixth section is very important, inasmuch as it contains the information a practical anatomist "obtained by dissection relative to the causation of other diseases by those of the digestive organs." Here we are first reminded of the well-known sympathy between the stomach and brain, and the consequent nervous complaints produced by it; from which Mr. A. infers, that "such a state of irritation of the sensorium may lay the foundation of an excitement of the vascular structure of the brain, and thus very frequently produce organic disease." We confess ourselves not perfectly satisfied with this conclusion. The cases given by our author in confirmation are highly valuable, but to us not satisfactory in the conclusions drawn from them. Six subjects, whose cases were attended to during life, were opened after death; one died with apoplectic symptoms, and five with hæmiplegia. In all, the liver was greatly diseased and the bowels also. In three there was inflammation of the membranes of the brain, with a good deal of water in the ventricles. In two no morbid appearance of the brain could be discovered. All these cases show us how much we have to learn, but by no means convince us that the sympathy with the sensorium continued so long as to alter the structure or vascular action of the brain in the manner described.

The sixth chapter contains two useful cases (one of them in the author himself) illustrative of the importance of attending to the digestive organs under symptoms little suspected to arise from diseases in those parts; and concludes with a polite acknowledgment of the hints received from Mr. Boodle, to whom Mr. Abernethy acknowledges

* This expression was addressed to the patient, but many readers, like ourselves, may be at a loss how to take it.

acknowledges himself obliged for being first directed to these considerations.

Such is the opinion we have formed of this work, of which we shall once more observe, that we regret it is not so good as it ought to be. At the same time we feel it our duty to acknowledge that the facts it contains are many of them too valuable to be laid by till one so much employed as the author, has leisure to arrange them for every class of readers. Another remark we would make relates to the language. There is throughout a playfulness of stile, and sometimes of words, which ill becomes physiology. Having made pretty free extracts we shall leave our readers to determine the justice of our objections to the style, but we think no one will justify the additional syllables in *morbific*, or *causation*, or conceive that our language is so barren as to render necessary the introduction of a term so undefined as *pseudo-syphilis*. These last are trifling objections, but as censors of medical literature we feel it our duty to mark them.

Two papers follow, one on diseases of the urethra, particularly that part which is surrounded by the prostate gland; the other, on the treatment of one species of nævi materni. They both contain good practical hints, but are not of a nature to be abridged.

---

*Remarks on the ineffective State of the Practice of Physic in Great Britain, with Proposals for its future Regulations and Improvement, and the Resolutions of the Members of the Benevolent Society of Lincolnshire.* By EDWARD HARRISON, M. D. *President of that Society;* F. R. A. S. *Edinburgh; of the Medical Society of London, &c.*

As this subject has for some time occupied the attention of a great number of professional gentlemen, it might appear inattentive to pass it over altogether. The principal thing deserving notice in the introductory part of the work is, that Apothecaries are not examined as well as Physicians and Surgeons. There cannot be a doubt that the regular apothecary would rejoice at any distinction which could be made between himself and the man who yesterday swept his shop, acquired the Latin names of a few drugs, and to day places his bottles with Arabic titles in his windows, talks more fluently on medicines, in proportion as he knows little, and promises with a boldness proportioned to the certainty, that he has no character to lose. All the various accusations brought by Dr. Harrison against the practitioners in his neighbourhood, may be true; but if they are, it is disgraceful to those who know it, that none of the perpetrators have been brought to condign punishment. It is a great mistake to suppose that any new laws are necessary for this. Half the public spiritedness which has blazed forth in this reforming zeal, would have taught these clamorous gentlemen that something may yet be done before it is necessary to apply to parliament. When all such measures have been ineffectually tried, then will be the time to apply for new laws, because we shall be able to plead practically, that the old ones are insufficient.

---

*Commentaries on the Lues Bovilla or Cow Pox. By* BENJAMIN MOSELEY, M. D. *Author of a Treatise on Tropical Diseases, of a Treatise on Coffee, and of Medical Tracts, &c. &c.*

WE should not have thought it necessary to take notice of this second edition, had it not been that our Journal affords, we believe, the only record of one of Dr. Moseley's cases, which he describes in the following words:

" Dr. Adams, of the Small Pox Hospital, has answered all objections made against the Cow Pox."

" I take the liberty to ask Dr. Adams, whether Elizabeth Harris, a young woman who had the Cow-pox two years ago, did not die of the confluent Small-pox, in the Small-pox Hospital, on the 7th of last month."

If the reader will take the trouble of perusing page 199 of our 14th vol. and also 353 of the same, he will see all the particulars of this case, and be convinced, as we were, that the woman never had Cow-pox.

